# Supplementary material for: Seminoma and Embryonal Carcinoma Footprints Identified by Analysis of Integrated Genome-Wide Epigenetic and Expression Profiles of Germ Cell Cancer Cell Lines
Source: PLoS One. 2014 Jun 2;9(6):e98330. doi: 10.1371/journal.pone.0098330 (PMC4041891; doi:10.1371/journal.pone.0098330)
Supplement: File S1 — ZIP file containing DMRforPairs output for significant regions. Please start from the html files. (ZIP) [file pone.0098330.s008.zip › figures/41781.pdf]

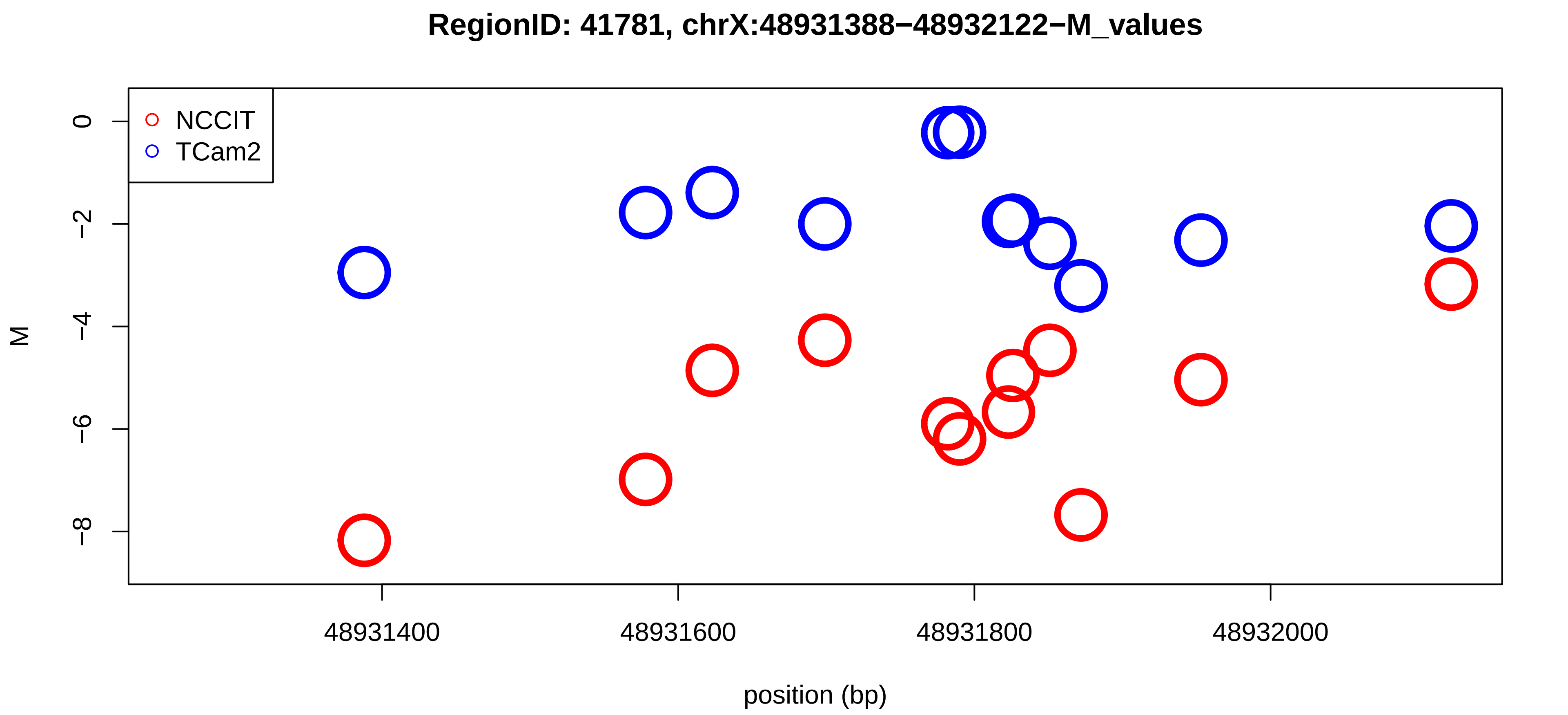
RegionID: 41781, chrX:48931388−48932122−M_values
NCCIT
TCam2
M
0
−2
−4
−6
−8
48931400
48931600
48931800
48932000
position (bp)

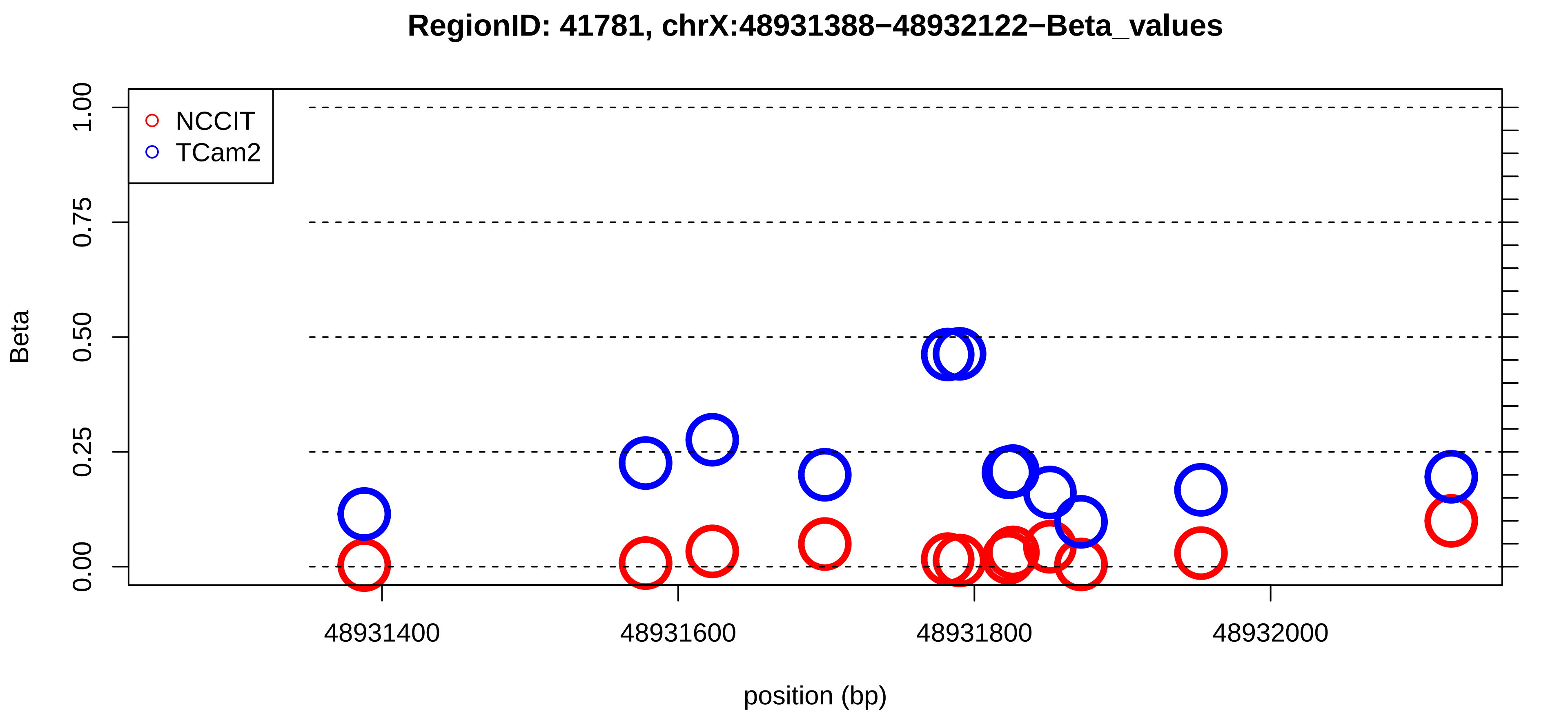
RegionID: 41781, chrX:48931388−48932122−Beta_values
NCCIT
TCam2
Beta
1.00
0.75
0.50
0.25
0.00
48931400
48931600
48931800
48932000
position (bp)

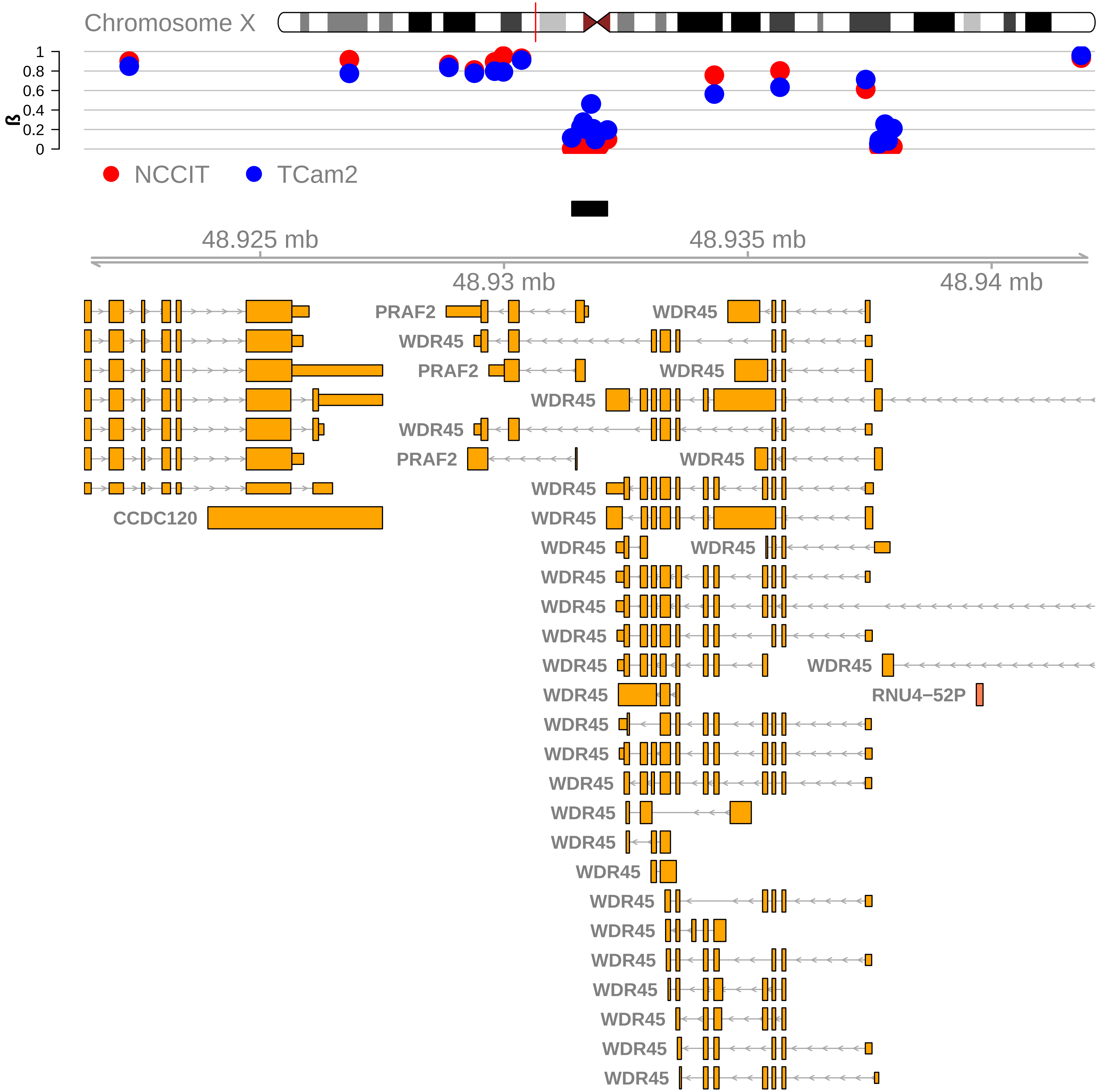
Chromosome X
1
0.8
0.6
0.4
0.2
0
β
NCCIT
TCam2
48.925 mb
48.935 mb
48.93 mb
48.94 mb
PRAF2
WDR45
WDR45
PRAF2
WDR45
WDR45
WDR45
PRAF2
WDR45
WDR45
CCDC120
WDR45
WDR45
WDR45
WDR45
WDR45
WDR45
WDR45
WDR45
RNU4−52P
WDR45
WDR45
WDR45
WDR45
WDR45
WDR45
WDR45
WDR45
WDR45
WDR45
WDR45
WDR45
WDR45
